# Localization of Two Sound Sources Based on Compressed Matched Field Processing with a Short Hydrophone Array in the Deep Ocean

**DOI:** 10.3390/s19173810

**Published:** 2019-09-03

**Authors:** Ran Cao, Kunde Yang, Qiulong Yang, Peng Chen, Quan Sun, Runze Xue

**Affiliations:** 1 School of Marine Science and Technology, Northwestern Polytechnical University, Xi’an 710072, China; 2 Key Laboratory of Ocean Acoustics and Sensing (Northwestern Polytechnical University), Ministry of Industry and Information Technology, Xi’an 710072, China

**Keywords:** sound source localization, compressive sensing, spatial filter, short hydrophone array, deep ocean

## Abstract

Passive multiple sound source localization is a challenging problem in underwater acoustics, especially for a short hydrophone array in the deep ocean. Several attempts have been made to solve this problem by applying compressive sensing (CS) techniques. In this study, one greedy algorithm in CS theory combined with a spatial filter was developed and applied to a two-source localization scenario in the deep ocean. This method facilitates localization by utilizing the greedy algorithm with a spatial filter at several iterative loops. The simulated and experimental data suggest that the proposed method provides a certain localization performance improvement over the use of the Bartlett processor and the greedy algorithm without a spatial filter. Additionally, the effects on the source localization caused by factors such as the array aperture, number of hydrophones or snapshots, and signal-to-noise ratio (SNR) are demonstrated.

## 1. Introduction

**Figure 1:**
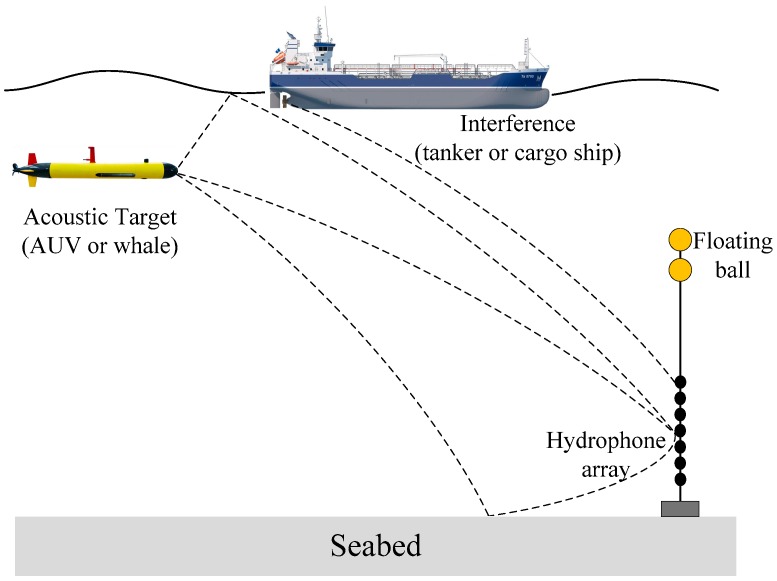
A schematic of an acoustic target (autonomous underwater vehicle or a whale) masked by a ship (a tanker or a cargo ship). The dashed lines represent the propagation paths from the sources to the hydrophone VLA.

Sound source localization with a short vertical line array (VLA) of hydrophones is challenging in the deep ocean (the array aperture is less than one-tenth the ocean depth) for the reason that a small aperture and a limited number of sensors cannot sample enough acoustic modes [1,2]. Furthermore, the source of interest can be obscured by a strong interfering source, which complicates the environment and limits the localization performance. For example, a target (an autonomous underwater vehicle or a whale) can be masked by a ship (a tanker or a cargo ship) near the VLA, as shown in Figure 1. And the aim of this paper is to propose a matched field processing (MFP) framework based on compressive sensing (CS) applied to the localization of two sources at single frequency with a short aperture array in the deep ocean.

There are a large number of published studies that propose methods for multiple source localization, which can generally be categorized into three types. The first type use the eigenvectors of the cross-spectral density matrix (CSDM) to estimate the positions of uncorrelated sources [3,4,5,6]. Collins et al. proposed a multivalued Bartlett processor based on the eigenvalue decomposition (EVD) of CSDM to estimate the moving source position [3]. Zurk et al. presented three motion mitigation techniques to improve the localization performance of moving targets [4]. The second type employs methods based on the Bayesian approach, utilizing the optimization of source parameters with the Markov Chain Monte Carlo sampling method [7,8,9,10,11]. Dosso and Wilmut provided two approaches utilizing the posterior probability density over the parameters of source position in uncertain shallow water [7]. Michalopoulou suggested that multiple sources can be localized using the maximum a posterior through Gibbs sampling [10]. The third type employ methods that use a spatial filter to subtract the contributions of the strongest source in the output of the modified matched field processor [12,13,14]. The CLEAN algorithm, which has been applied in astronomy to denoise images, was introduced for eliminating strong interferences to localize a weak source [12]. In most cases, this procedure can be applied repeatedly until each source’s localization is estimated. Neilsen reported am iterative inversion approach based on simulated annealing algorithms with a spatial matrix filter to locate multiple sources [13].

Recent studies have been carried out using the compressive sensing algorithms, which can be applied to underwater acoustics to achieve results with a high resolution [15]. The number of sources is far less than the number of possible positions, resulting in a sparse solution set. Therefore, the localization problem can be reformulated as a problem of obtaining the sparse solution of the source positions with CS methods. Liu et al. chose the basis pursuit de-noising method to solve the sparse representation problem with the homotopy approach and coordinate descent search to increase efficiency [16]. Zhang et al. combined the orthogonal matching pursuit (OMP) algorithm with efficient matched phase coherent MFP to solve the broadband multiple source localization problem in shallow water [17]. Gemba et al. investigated the performance of compressive MFP in shallow water [18]. Furthermore, Gemba et al. applied sparse Bayesian learning for the localization of robust multiple sources with SWellEx-96 data [19,20].

To date, little attention has been paid to the problem of multiple source localization with a short array in the deep ocean. The importance and originality of this study is that it explores the feasibility of two-source localization using a short array in the deep ocean, which is different from the above localization methods. In most cases that multiple source localization methods were applied in shallow water, the results could be obtained directly without many iterations. This is because enough acoustic modes can be sampled with the aperture of the array approximating to the ocean depth, which is impossible in the deep ocean. In practice, the cost of a large array is high and the deployment and maintenance of it are difficult in the deep ocean. Therefore, a short array is a more attractive choice for source localization in the deep ocean. Based on the depth-dependent interference between direct and surface-reflected arrivals, Mccargar and Zurk proposed a depth-based signal separation (DBSS) method using a short VLA on the reliable acoustic path, where the depths of different sources can be passively estimated by a modified Fourier transform to the power output of the vertical beamformer [21]. Moreover, Kniffin et al. analyzed the factors impacting the performance of the DBSS and introduced a depth estimation method utilizing the null spacing in the interference structure [22]. However, this method requires sources that have been moving for a relatively long time period to implement DBSS.

This paper proposes a new methodology for estimating two-source locations, employing one greedy method in an iterative manner. The greedy method is one type of recovery algorithms to obtain the sparse signals [23]. This method shows that the source localized directly by conventional methods may not be the real target. Furthermore, the technique has no need for the motion of sources. Compared to conventional methods, this technique can estimate the target with a high resolution, owing to the characteristics of CS theory. It is beyond the scope of this study to examine the robustness of this method to the environmental uncertainties of the ocean, which would affect the performance of this technique.

The remaining part of the paper proceeds as follows: Section 2 introduces the new processor for solving the reformulated sparse representation problem in a deep ocean scenario. In Section 3, the proposed method is applied to the simulated and experimental data and the factors affecting the localization performance are also presented. Finally, Section 4 gives a brief summary of the results, the limitations of the proposed method and future studies.

## 2. Source Localization Based on CS with Matrix Filter

### 2.1. Source Localization Using MFP and Spatial Filter

An oceanic waveguide is considered in this paper where the acoustic field $y_{k}(r_{k})$ is induced by one source at single frequency in the coordinates $r_{k}=\left(r_{k},z_{k}\right)$, where the index k of $y_{k}$ represents the received signals from the *k*th source. A VLA consists of *N* sensors, which is used for receiving signals. The data at the VLA can be expressed as

(1) $$y=\sum_{k=1}^{K}y_{k}(r_{k})=\sum_{k=1}^{K}S_{k}G_{k}(r_{k})+N$$

where *K* is the number of sources, N represents the uncorrelated additive ambient noise across frequencies at the VLA, $G_{k}\left(r_{k}\right)$ is the Green function vector, which represents the transferring function from the *k*th source at position $r_{k}$ to the VLA, and $S_{k}$ is the frequency spectra of the *k*th source. The CSDM is estimated by the spectral snapshots $y_{l}$:

(2) $$R=\frac{1}{L}\sum_{l=1}^{L}y_{l}y_{l}^{H},$$

where the index l represents the *l*th snapshot, *L* is the number of the snapshots and the superscript H denotes the conjugate transpose operation.

In MFP, replica field refers to the received signal vectors at the VLA, which are calculated by the acoustic models. One source in the replica field can be assumed to be at position ${\hat{r}}_{s}=\left({\hat{r}}_{s},{\hat{z}}_{s}\right)$, and the acoustic field can be expressed as $G\left({\hat{r}}_{s}\right)$. The normalized replica vector is $w\left({\hat{r}}_{s}\right)=G\left({\hat{r}}_{s}\right)/\left|G\left({\hat{r}}_{s}\right)\right|$. Then, the conventional Bartlett processor can be written as:

(3) $$B_{B}=w^{H}\left({\hat{r}}_{s}\right)Rw\left({\hat{r}}_{s}\right).$$

By computing Equation (3) for all the possible source positions, one can make an ambiguity surface, where the main lobe is the position of the source with the largest power.

The spatial matrix filter is applied to suppress the interference or multiple source localization. The filtered received signals can be expressed as

(4) z=Py,

where P is an N×N matrix of complex numbers. Two major design methods for the matrix filter exist, where the passband fidelity is minimized subject to controlled stopband attenuation and vice versa. This problem, which is treated as a semidefinite program (SDP), can be solved by SEDUMI in the CVX toolbox [24]. However, the computation complexity of solving an SDP is high. Mantzel et al. proposed a method named by ROMOLO for multiple source localization with a simpler matrix filter, where a low-rank projection matrix is constructed by minimizing the average stopband attenuation subject to the controlled passband fidelity [25]. This method can enhance the computation efficiency greatly and be applied to the multiple source localization in this work. First, the source positions from ${\hat{r}}_{1}$ to ${\hat{r}}_{Q}$ are estimated in sequence, where *Q* is the maximum iterative number. Then, the correlation matrix at the *q*th loop is constructed as follows:

(5) $$M_{q}=G_{q}(ar_{q})G_{q}{\left(ar_{q}\right)}^{H},$$

where $ar_{q}$ is an elliptical area surround the estimated position $r_{q}$ and q is the iteration index. For the estimated source position in the *q*th loop, the ellipse area adopted for a spatial filter can be expressed as $ar_{q}=\left\{\left(r,d\right):D_{q}<1\right\}$, where r and d represent the range and depth of the search grids, respectively. The distance $D_{q}$ between the estimated position and the grid point position is constructed as follows:

(6) $$D_{q}=\sqrt{\left(\frac{r-r_{q-1}}{r_{w}}\right)+\left(\frac{d-d_{q-1}}{d_{w}}\right)},$$

where $r_{q-1}$ and $d_{q-1}$ are the estimated range and depth in the previous loop, and the index w represent the weight coefficient in range and depth. Then, EVD is applied to the correlation matrix to extract the eigenvectors $U_{n}$, *n* = 1, …, *N*. Lastly, the projection matrix is constructed by the first *nr* (nulling rank) columns of the eigenvector matrix:

(7) $$P=I-U_{nr}U_{nr}^{H}.$$

where $U_{nr}$ represents the first *nr* eigenvectors, $U_{nr}=\left[U_{1},U_{2},\ldots ,U_{nr}\right]$.

In a word, the above methods are the conventional methods for source localization. Then, a new method combined the CS theory and matrix filter is introduced.

### 2.2. Localization Method Combined with CS and Spatial Filter

In real ocean environments, the number of sources is far less than that of potential positions. Thus, a sparse solution can be estimated for the source localizations with a large size dictionary of replicas in CS. For simplification, the notations are as follows:

$$\begin{array}{l} A=\left[a_{1},a_{2},...,a_{M}\right],\\ a_{m}=G_{m}({\hat{r}}_{s})/{\left\|G_{m}({\hat{r}}_{s})\right\|}_{2},\\ x_{k}=S_{k}{\left\|G_{k}({\hat{r}}_{s})\right\|}_{2}, \end{array}$$

where M is the number of research grids. Then, the array data can be rewritten as

(8) y=Ax+N,

where $A\in {\mathbb{C}}^{N\times M}$ is the replica matrix for the potential positions, while $x={\left[x_{1},x_{2},...,x_{M}\right]}^{T}$ and y denote a signal vector and the measurement vector, respectively. The source positions correspond to the nonzero elements in x. The estimation of the sources can be expressed as the $l_{0}$-norm minimization problem:

(9) $$\arg\min{\left\|x\right\|}_{0},s.t.{\left\|y-Ax\right\|}_{2}<\varepsilon ,$$

where the objective function ${\left\|x\right\|}_{0}$ is nonconvex and ε is the error threshold. The techniques used to solve this problem based on the sparse theory can be categorized into four major types: Greedy algorithms, convex relaxation techniques, iterative algorithms, and statistic sparse recovery techniques [23]. The computation complexity of convex relaxation techniques (e.g., Basis Pursuit De-noising) and statistic sparse recovery (e.g., sparse Bayesian learning) is larger than that of greedy algorithms (e.g., OMP). The application of iterative algorithms is limited. Therefore, one of the greedy algorithms called the sparsity adaptive matching pursuit (SAMP) algorithm is introduced in this paper [26]. The greatest advantage of SAMP is that the signal can be reconstructed without the knowledge of the sparsity, which is hard to be obtained practically. Moreover, a regularized step is added into the SAMP algorithm to improve the reconstruction performance and decrease the computational power [27]. The regularized SAMP algorithm (RAMP) is summarized in Algorithm 1.

**Algorithm 1** Regularized sparsity adaptive matching pursuit (RAMP) algorithm [27]Input: y, A, S (step size), IM (maximum number of iterations), $\varepsilon_{\min}$ (a positive parameter)Initialization: 
$\hat{x}=0$, $rv_{1}=y$, Λ=∅, L=S (size of the finalist), t=1 (iteration index), j=1 (stage index)While 1≤t≤IM and ${\left\|rv_{t}\right\|}_{2}>\varepsilon_{\min}$
$u=\left|A^{H}rv_{t-1}\right|$,
J=max(u,L) Regularize the process to find subset $J_{0}$, $J_{0}\in J$
|u(i)|≤2|u(j)|,i,j∈J,  Choose $J_{0}$ with the maximum energy ${\left\|u_{J_{0}}\right\|}_{2}$
$\Lambda_{t}=\Lambda_{t-1}\cup J_{0}$
${\hat{x}}_{t}={\left(A_{\Lambda_{t}}^{H}A_{\Lambda_{t}}\right)}^{-1}A_{\Lambda_{t}}^{H}y$
$F=\max\left(\left|{\hat{x}}_{t}\right|,L\right)$
$rv_{t}=y-A_{F}^{H}A_{F}y$ If ${\left\|rv_{t}\right\|}_{2}\geq {\left\|rv_{t-1}\right\|}_{2}$   j=j+1, L=j×S else   Λ=F, $rv_{t}=rv_{t-1}$, t=t+1 End if End WhileOutput:
$\hat{x}=A_{F}^{H}y$


The solution of Equation (9) can be expressed as [18,23]

(10) $$\hat{x}=\arg\underset{m}{\max}\left|rv^{H}a_{m}\right|,$$

where $rv(\omega_{f})$ is the residual vector and |·| is the absolute value operator. Note that the sparse model can be also extended to multiple snapshots to improve the localization performance. Further, the normalization problem in Equation (9) is transformed into [18]

(11) $$\arg\min\sum_{j=1}^{M}{\left\|X_{j}\right\|}_{2},s.t.{\left\|Y-AX\right\|}_{F}^{2}<\varepsilon ,$$

where ${\left\|\cdot \right\|}_{F}$ denotes the Frobenius norm operator and $Y\in {\mathbb{C}}^{N\times L}$ includes *L* snapshots for a stationary source. This problem is also called the multiple measurement vectors problem, which can be solved by the extension of the aforementioned four major methods. Because matrix X is row sparse, the RAMP algorithm can be extended by summing up the $l_{2}$-norm of the each row in the matrices, such as u, $rv_{m}$ in Algorithm 1. Additionally, for the comparison with the Bartlett processor, the average power at position ${\hat{r}}_{k}=\left({\hat{r}}_{k},{\hat{z}}_{k}\right)$ is regarded as [18]

(12) $$P_{R}\left({\hat{r}}_{k}\right)={\left\|{\hat{x}}_{k}\right\|}_{2}^{2}/L={\left\|a_{k}^{H}Y\right\|}_{2}^{2}/L,$$

where the row vector $a_{k}$ corresponds to ${\hat{r}}_{k}$ in the replica field and ${\hat{x}}_{k}$ corresponds to *k*th value in the solution of Equation (10). The corresponding ambiguity surface can be obtained by computing the output at each position ${\hat{r}}_{s}$ in the replica fields, $B_{R}\left({\hat{r}}_{s}\right)=10\log_{10}\left(P_{R}\left({\hat{r}}_{s}\right)/\max\left(\left[P_{R}\left({\hat{r}}_{1}\right),\ldots P_{R}\left({\hat{r}}_{s}\right),\ldots P_{R}\left({\hat{r}}_{M}\right)\right]\right)\right)$.

The approach for the localization of two sources is to estimate the peak of the ambiguity surface as the most prominent source using RAMP and repeatedly apply the matrix filter to suppress its contribution. This method is similar to ROMULO. However, one of the unknown sources can be localized accurately in each loop within ROMULO, whereas the contribution of the already estimated sources are rejected in the latter loop. This is difficult to obtain in a complex ocean environment. The proposed method is based on the RAMP algorithm and matrix filter. Herein it is referred to as RAMPMF and summarized in Algorithm 2. Additionally, the performances and comparisons of the RAMP algorithm and the RAMPMF algorithm are presented in the next section. The originality of this study is combining the RAMP method and the matrix filter to localize two sources in an iterative way.

**Algorithm 2** RAMP algorithm combined with matrix filter (RAMPMF) algorithmInput:
y, A, $\left(r_{w},d_{w}\right)$ (elliptical norm weighting coefficients), nr (nulling rank)
Q (maximum iterative number), S (step size), $\varepsilon_{\min}$ (a positive parameter)Initialization:
$\hat{x}=0$, rv=y, Λ=∅ (parameters used in the RAMP algorithm), P=I (projection matrix) RAMP search for a source position $r_{0}=\left(r_{0},d_{0}\right)$ and ${\hat{x}}_{0}$
for q = 1: Q
$D_{q}=\sqrt{\left(\frac{r-r_{s_{q-1}}}{r_{w}}\right)+\left(\frac{d-d_{s_{q-1}}}{d_{w}}\right)}$
$ar_{q}=\left\{\left(r,d\right):D_{q}<1\right\}$
$M_{q}=G_{q}(ar_{q})G_{q}{\left(ar_{q}\right)}^{H}$
$M_{q}=U\sum^{2}U^{H}$ if q = 1, P=I else $P_{q}=I-U_{nr}U_{nr}^{H},$ endif
$y_{q}=Py_{q-1}$
$A_{q}=PA_{q-1}$ RAMP search for a new source position $r_{q}=\left(r_{s},d_{s}\right)$, ${\hat{x}}_{q}$ End forOutput: $\left[r_{1},\ldots ,r_{Q}\right]$ and $\left[{\hat{x}}_{1},\ldots ,{\hat{x}}_{Q}\right]$


It should be noted that the localized source positions may be different in each iterative loop. Therefore, the correlation between the different localizations is utilized to search for the similarity among the different positions. The correlation coefficient can be expressed as cosine law:

(13) $$coef\left(r_{i},r_{j}\right)=\frac{r_{i}r_{j}}{{\left\|r_{i}\right\|}_{2}{\left\|r_{j}\right\|}_{2}},$$

where $r_{i}=\left(r_{i},d_{i}\right)$ and $r_{j}=\left(r_{j},d_{j}\right)$ are the source positions obtained in the *i*th and *j*th iterative, respectively. The range $r_{i}$ is transformed into the same order of magnitude with the depth $d_{i}$. The degree of correlation is high when the coefficient approaches 1.

However, the threshold of the correlation coefficient is hard to define for the reason that there are many values close to one, such as 0.993 and 0.99. A new correlation coefficient between $r_{i}$ and $r_{j}$ is defined as:

(14) $$coef\left(r_{i},r_{j}\right)=\sqrt{2}\frac{\left|d_{i}-d_{j}\right|}{d_{i}+d_{j}}+\sqrt{2}\frac{\left|r_{i}-r_{j}\right|}{r_{i}+r_{j}}.$$

Contrary to Equation (13), the degree of correlation is high when the coefficient in Equation (14) approaches zero. In general, the threshold is set as 0.1. For example, the coefficient of $r_{1}=\left(8220,48\right)$ and $r_{2}=\left(9000,67\right)$ in Equation (13) is 0.9938, whereas that in Equation (14) is 0.2977 more than the threshold of 0.1. Then, the number of potential sources is determined by the correlation coefficient of the different localizations in the unique circle $\left[r_{z},r_{z+1}\right]$, which corresponds to the source positions. The average power per snapshot provided by the RAMPMF algorithm is calculated by $P_{RM}\left({\hat{r}}_{k}\right)=\frac{1}{2}\sum_{m}^{m+1}{\left\|{\hat{x}}_{k}\right\|}_{2}^{2}/L$, where *k* is chosen from z to z+1, where z represents the index of one cycle. The corresponding ambiguity surface can be obtained by $B_{RM}\left({\hat{r}}_{s}\right)=10\log_{10}\left(P_{RM}\left({\hat{r}}_{s}\right)/\max\left(\left[P_{RM}\left({\hat{r}}_{1}\right),\ldots P_{RM}\left({\hat{r}}_{s}\right),\ldots P_{RM}\left({\hat{r}}_{M}\right)\right]\right)\right)$.

## 3. Numerical Results

### 3.1. Simulated Data

**Figure 2:**
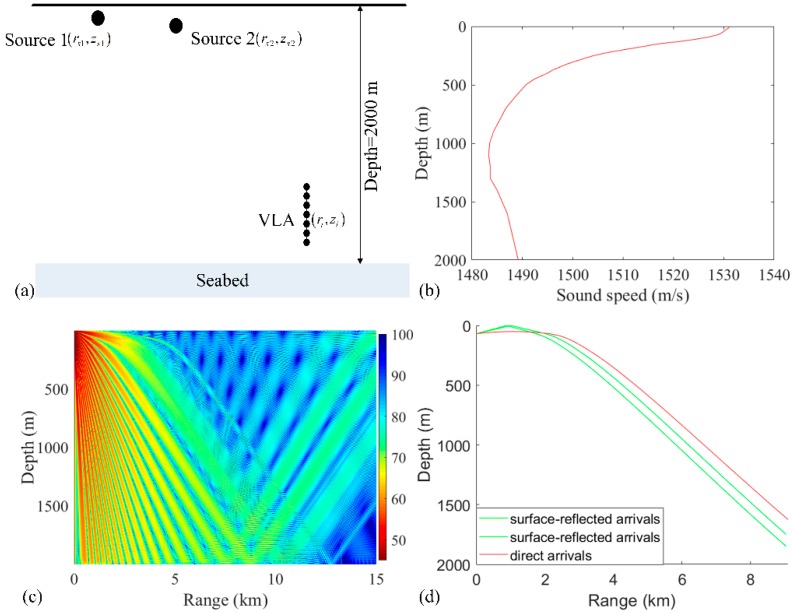
(**a**) Schematic of a source propagating to the vertical line array (VLA) in the deep water. $\left(r_{si},z_{si}\right)$ and $\left(r_{i},z_{i}\right)$ are the positions of a towed source and one hydrophone of the VLA, respectively. (**b**) The sound speed profile (SSP) was measured by a conductivity–temperature–depth (CTD) probe in the experiment. (**c**,**d**) Calculated TL and eigenrays using BELLHOP with the SSP shown in (**b**) at 187 Hz and a source at a depth of 100 m.

The RAMPMF method is applied to a simulated dataset and experimental data to compare the different processors and demonstrate the performance. A VLA was used for this simulation including 32 hydrophones, which were deployed from 1735 to 1859 m with a 4 m interval. Two sources both at frequency 187 Hz were treated as the target sources. A flat bathymetry was at a depth of 2000 m. The schematic geometry of the acoustic propagation in this scenario is shown in Figure 2a. The sound speed profile in the water was measured by a conductivity–temperature–depth (CTD) probe near the VLA in the experiment, as shown in Figure 2b. Figure 2c shows that the transmission loss (TL) received on the VLA below 1700 m is less than that on the sea surface in the range from 3 to 9 km. This is because the main energy received in the VLA is contributed by the direct arrivals and surface-reflected arrivals, as shown in Figure 2d. In fact, the ranges of interest are from 1 to 15 km and the depths of interest are from 0 to 400 m in the deep ocean. One reason for this is that the propagation path of the sound wave is curved due to the depth-dependent sound speed profile, as shown in Figure 2d. These characteristic limits the direction of the sources. The elevation angles of the region of interest are between endfire and boresight.

In the numerical simulation, the ray model BELLHOP [28] was used to compute the replica vectors at a single frequency of 187 Hz. The corresponding half-wavelength approximates the spacing of the VLA. The sound speed of the compressional wave as well as the density and attenuation coefficient of the sediment were 1550 m/s, 1310 kg/m^3^, and 0.15 dB/λ, respectively. The range-dependent environmental factors, such as sound fluctuation caused by internal waves, were neglected. Therefore, the theory of reciprocity [29] was applied to this scenario to reduce the computation, and each of hydrophones of the VLA was treated as a virtual source to obtain the replicas. The search area was (0.4 km, 15 km) × (0 m, 400 m) with a range and depth discretization of 20 and 1 m, respectively.

A two-source scenario (sources 1 and 2) is considered in the abovementioned environment. The positions of source 1 and source 2 are (3 km, 70 m) and (6 km, 10 m), respectively. The Gaussian white noise, which is identically distributed, is added into the signals to obtain:

(15) $$Y=a_{1}x_{1}^{T}+a_{2}x_{2}^{T}+N,$$

where $a_{i}$ represents the Green function between the *i*th (*i* = 1, 2) source and the VLA. Each $x_{i}\in C^{L}$ is the complex amplitude vector for the *i*th source, which is chosen independently from a uniform distribution for each snapshot to construct the sources. Each snapshot has the same signal-to-noise ratio (SNR). The definition of the average SNR at one hydrophone of the VLA for the source is shown below [28,29]:

(16) $$SNR=10\log_{10}E\left\{\sum_{i}{\left\|a_{i}x_{l}\right\|}_{2}^{2}\right\}/E\left\{{\left\|N_{l}\right\|}_{2}^{2}\right\},$$

where $x_{l}$ is the complex magnitude for the *l*-th snapshot. In addition, the SNRs in the simulations were the ratios of the summation of time domain signals at 3 and 6 km to the white noise. The source magnitudes are determined with the assumption that the source 1 to source 2 ratio (SSR) is 5 dB. The SSR at different snapshot is defined as:

(17) $$SSR=20\log_{10}\left({\left\|a_{1}x_{l}^{T}\right\|}_{F}/{\left\|a_{2}x_{l}^{T}\right\|}_{F}\right).$$

For the spatial filter, the elliptical norm weighting coefficients in range and depth ($r_{w}$ and $d_{w}$) are assumed to be 72 and 8 m, respectively. The first five (nulling rank) columns of the eigenvector matrix are selected for constructing the projection matrix. In addition, the maximum iterative number is assumed to be 20. To analyze the performance of the RAMPMF method, the estimated range and source errors are expressed as:

(18) $$e_{r}=\left|\left(r_{e}-r_{S}\right)/r_{S}\right|,e_{d}=\left|\left(d_{e}-d_{S}\right)/d_{S}\right|,$$

where $r_{e}$ and $d_{e}$ are the estimated source range and depth of the peak in the ambiguity surface, $r_{S}$ and $d_{S}$ are the real source range and depth, respectively. In this two-source scenario, the estimated localization error for each source is computed. The accurate localization (estimation) of one source means that the estimated range and depth errors $e_{r}$ and $e_{d}$ are both below 10% in the configuration in this study as it can be seen below. Similarly, the accurate localization (estimation) of two sources refers to the accurate estimation to each source in the same realization. Especially, the Bartlett processor is always used as the benchmark for the comparisons between different methods in the underwater acoustics [3,6,12,18].

**Figure 3:**
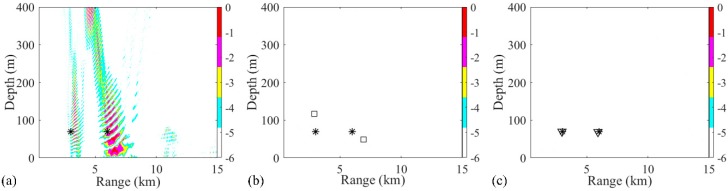
Ambiguity surfaces from the simulated data at 187 Hz for the localization of two sources using (**a**) the Bartlett processor, (**b**) the RAMP method, and (**c**) the RAMPMF method. True source positions and the positions estimated by the RAMP and RAMPMF methods are represented as black asterisks, squares, and inverted triangles, respectively.

In the first two-source scenario, the positions of source 1 and source 2 are set as (3 km, 70 m) and (6 km, 70 m), respectively. The SNR at each snapshot is defined as 5 dB, and the number of snapshots is set to 28. The ambiguity surfaces (AMSs) of different processors are shown in Figure 3. The range and depth coordinates are reduced to highlight the localized grids. The true source positions and the positions estimated by the RAMP and RAMPMF methods are represented as black asterisks, squares, and inverted triangles, respectively. Strong sidelobes exist in the AMS of the Bartlett processor in Figure 3a. The two largest peaks in the AMS are (3.38 km, 70 m) and (7.32 km, 49 m), which are deviated from the true sources. Based on Equation (18), the range and depth errors corresponding to these points are (12.67% and 0%) and (22% and 30%), respectively. The estimated source positions obtained by the RAMP method are (2.9 km, 117 m) and (6.92 km, 49 m), as shown in Figure 3b, which are the two grids corresponding to the two maximum values in the output ${\hat{x}}_{s}$. The range errors are 3.33% and 15.3%, whereas the depth errors are large. Even for the given number of sources, the localization performance of RAMP is still poor. Part of the reason for this is that the CS method characteristically generates few solutions. The sparse solutions can be the same pseudo peaks that exist in the AMS of the Bartlett processor, as shown in Figure 3a, and the values at the true positions are zero. Another possible reason is that the interference formed by the single-frequency source results in pseudo peaks at large distances from the true sources, such as the pseudo peaks that appear at (4 km, 400 m) in Figure 3a. The output ${\hat{r}}_{s}$ of RAMPMF is shown in Table 1. A repeated circle ((2.98 km, 70 m) and (5.92 km, 69 m)) can be obtained in the iterative loop. The length of this circle is 2, representing two localized sources obtained by the RAMPMF method. The range and depth errors are (0.67%, 0%) and (1.33%, 1.43%), respectively. Based on Equation (12), the ambiguity surface can be the summation of ${\hat{x}}_{s_{3}}$ and ${\hat{x}}_{s_{4}}$, corresponding to the indices in the localization loops. In 500 Monte Carto realizations, the probability of the accurate estimation of two source positions was found to be 93.4%.

**Figure 4:**
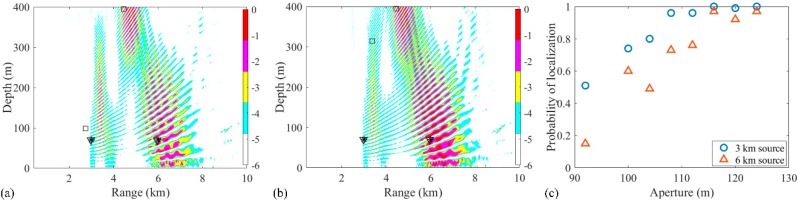
(**a**,**b**) Ambiguity surfaces generated by different realizations for a VLA from 1735 to 1835 m with fixed spacing using the RAMPMF method, compared to the results shown in Figure 3. The true source positions and the positions estimated by the RAMP and RAMPMF methods are represented by black asterisks, squares, and inverted triangles, respectively; (**c**) The probability of obtaining an accurate estimation of the source depth with different apertures for 3 and 6 km sources.

Due to the sparse solutions shown in Figure 3, the results obtained from the RAMP and RAMPMF processors, which are represented as squares and inverted triangles, would be placed in the same figure generated by the Bartlett processor. Then, the effects of factors such as the array aperture, number of receivers, number of snapshots, and SNR on the localization results could be investigated. Only one factor was considered at a time, with the other factors remaining the same in the environment. First, the dependence of the localization on the array aperture was examined with a fixed spacing. The VLA was spaced between 1735 and 1835 m with a fixed spacing 4 m and the number of hydrophones was 26. The other parameters, such as SNR and SSR, remained the same. Similar to the results in Figure 3, it is shown in Figure 4a,b that the localization results by the RAMPMF method are better than the results of the two other processors. The probability of obtaining an accurate estimation of source depths with different apertures for 3 and 6 km sources is shown in Figure 4c. A clear trend is shown, indicating that the localization performance can be improved by increasing the aperture of the VLA. And the probability of two-source localization above 0.9 can be achieved with the array length greater than 114 m, which is only 5.7% of the total water depth. On the other hand, the probability of depth estimation for an aperture less than 100 m is relatively low in the 2000-meter deep water. A possible reason for this is that is difficult to obtain an accurate solution with the measured vector of limited length and a large sensing matrix using CS theory. Besides, the columns of the sensing matrix are partially correlated, introducing error to the result.

**Figure 5:**
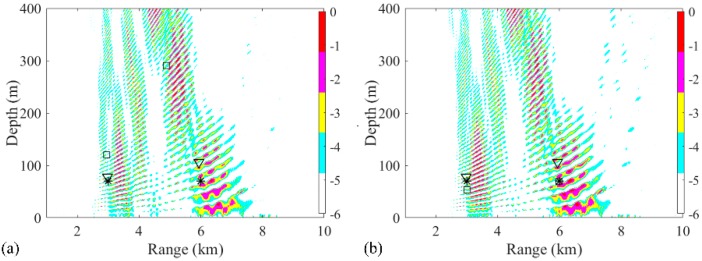
(**a**,**b**) Ambiguity surfaces generated by different realizations with the elements of the VLA 8 m apart (from 1735 to 1859 m), compared to the results shown in Figure 3. True source positions and the positions estimated by the RAMP and RAMPMF methods are represented as black asterisks, squares, and inverted triangles, respectively.

**Figure 6:**
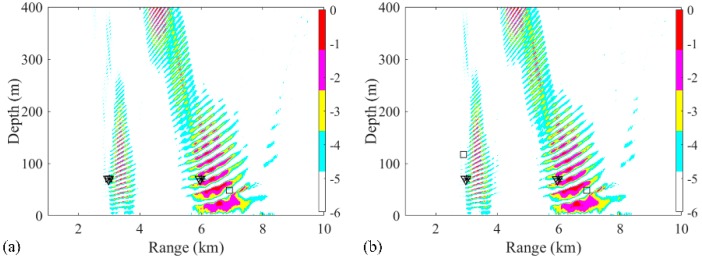
(**a**,**b**) Ambiguity surfaces generated by different realizations with the elements of the VLA 2 m apart (from 1735 to 1859 m). True source positions and the positions estimated by the RAMP and RAMPMF methods are represented as black asterisks, squares, and inverted triangles, respectively.

The second studied factor was the number of receivers, which corresponds to the element spacing with a given array aperture. In this environment, the number of receivers decreases to 16 and the spacing is approximately 8 m. The other parameters stay the same, such as SNR and SSR. The source localization performance by RAMPMF degrades, as shown in Figure 5, whereas the main lobe in the AMS obtained by the Bartlett processor is similar, as shown in Figure 3a, except for the increasing sidelobes. Therefore, using fewer hydrophones degrades the performance seriously for all the methods. When the spacing is about the same as the wavelength of the 187 Hz single-frequency signal, pseudo peaks (such as the grating lobe in the beam pattern) appear, and the true source positions are submerged in the AMS. On the other hand, the performances of different methods with 2 m spacing are shown in Figure 6. It is indicated that the RAMPMF method performs well with a spacing that is no more than half the wavelength.

**Figure 7:**
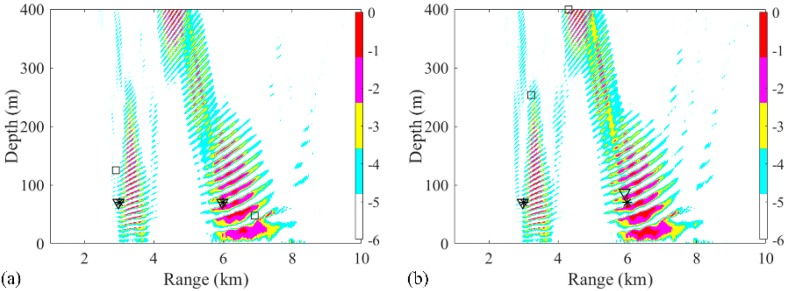
(**a**,**b**) Ambiguity surfaces generated by different realizations with 14 snapshots using the RAMPMF method, compared to the results shown in Figure 3. True source positions and the positions estimated by the RAMP and RAMPMF methods are represented as black asterisks, squares, and inverted triangles, respectively.

**Figure 8:**
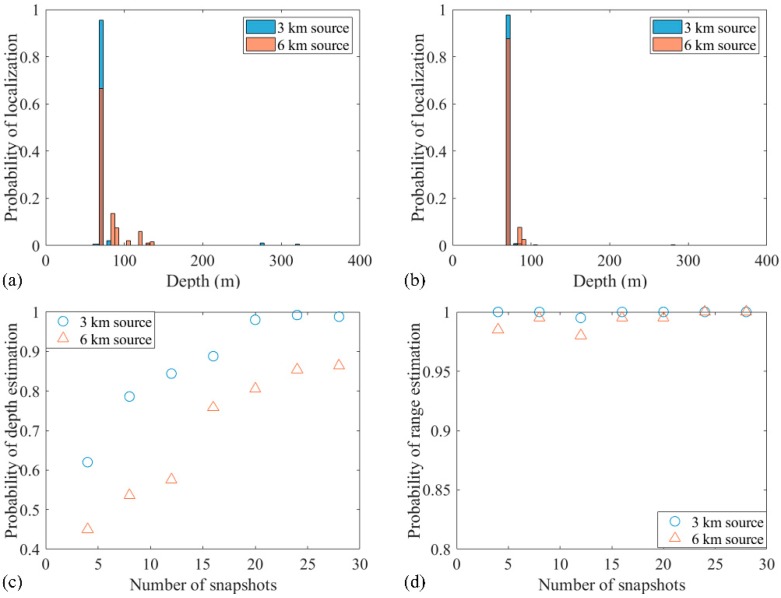
Effect of the number of snapshots on the localization for the two-source case with the signal-to-noise ratio (SNR) = 5 dB and the source 1 to source 2 ratio (SSR) = 5 dB: (**a**) 14 snapshots; (**b**) 28 snapshots. Probabilities of (**c**) depth estimation and (**d**) range estimation with different snapshots.

Also, the number of snapshots affected the localization performance. To determine this result, only the snapshot is changed while the SNR is 5 dB, the SSR is 5 dB, and the length of the 32 element VLA is 124 m. The source localization with 14 snapshots is shown in Figure 7. The probabilities of accurate estimation of 3 and 6 km sources in 500 realizations were 95.8% and 69.8%, respectively. The probability distribution graphs of accurate localization for 3 and 6 km sources with 14 and 28 snapshots are shown in Figure 8a,b, respectively. Also, the probability of accurate depth estimation increases with an increase in the number of snapshots, as shown in Figure 8c. The individual depth estimation error for each source is represented. It is demonstrated from the result that the depth error of further sources is larger than that of closer sources for any number of snapshots. Further, the probability of accurate range estimation versus the number of snapshots is also demonstrated in Figure 8d. Compared with the probability of depth estimation, the estimated ranges for sources at 3 and 6 km were very accurate. The result also indicates that more snapshots result in more accurate localization. 

**Figure 9:**
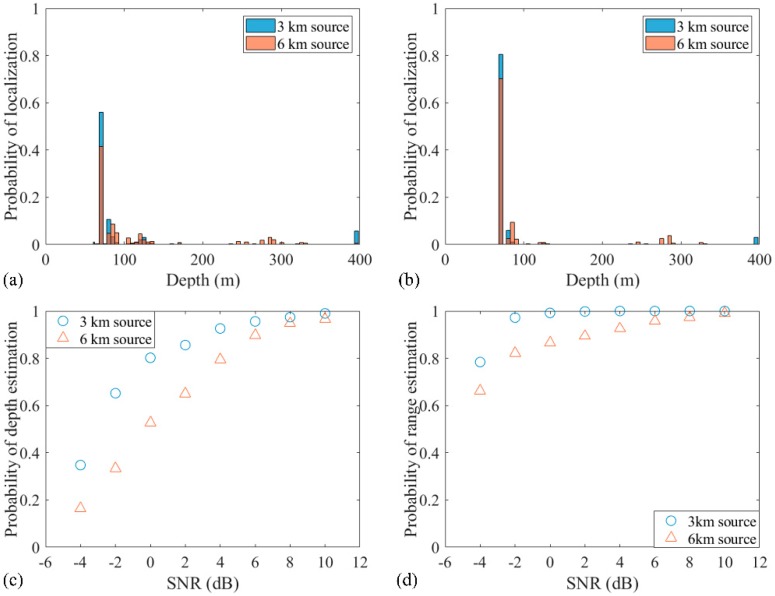
Effect of SNRs on the localization for the two-source case with SSR = 5 dB: (**a**) −2 dB and (**b**) 2 dB. Probabilities of (**c**) depth estimation and (**d**) range estimation with different SNRs.

The localization performance versus SNR is shown in Figure 9. The SNR is changed with the increment of noise power whereas SSR is maintained at 5 dB. The probability distribution graphs of accurate localization for 3 and 6 km sources with −2 and 2 dB SNRs are shown in Figure 9a,b, respectively. It is clear that the probability of localization for the 3 and 6 km sources increases with the increase of the SNR to 2 dB. Also, the probabilities of depth and range estimations increase with higher SNRs, as shown in Figure 9c,d. Still, the range estimation of the RAMPMF method is more robust than the depth estimation when the SNR changes from −4 dB to 10 dB. Similar to the analysis for the snapshots, the RAMPMF method was found to perform better with respect to a higher SNR.

**Figure 10:**
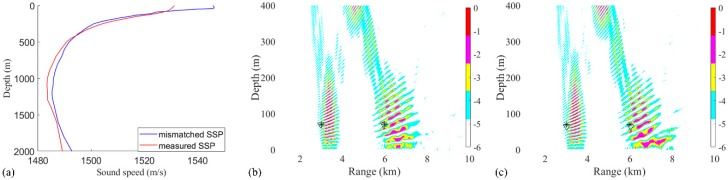
**(a)** The measured and mismatched SSPs are represented as the red line and blue line, respectively; (**b**) the ambiguity surfaces calculated by a Bartlett processor and the RAMPMF method using the measured SSP; (**c**) the ambiguity surfaces calculated by a Bartlett processor and the RAMPMF method using the mismatched SSP.

**Figure 11:**
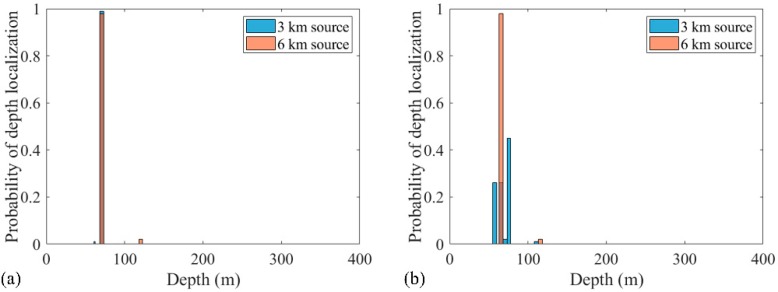
The probabilities of depth estimation within 500 realizations using (**a**) the measured SSP and (**b**) the mismatched SSP.

The effect of SSP on the performance was also examined. Another SSP was chosen for computing the replicas, as shown in Figure 10a. The parameters, such as source positions, SNR, and SSR, were the same as them in the Figure 3. The positions of two sources estimated by RAMPMF method using the measured SSP are (2980 m, 70 m) and (5960 m, 70 m) in one realization in Figure 10b. The positions of two sources estimated by RAMPMF method using the mismatched SSP are (3020 m, 73 m) and (6040 m, 64 m) in the same realization in Figure 10c. Additionally, 500 Monte Carto realizations were conducted using the mismatched SSP, the probability of the accurate estimation of two source positions was found to be 73%, less than the probability of 93.4% using the measured SSP. However, the distributions of the depths of two sources are shown in Figure 11. The result showed that most of the estimated depths were changed from 70 to 64 m or 73 m for 3 km and 6 km sources. The performance influenced by the mismatched SSP is not as much as it is affected by SNR. The reason is that main energy received in the VLA is contributed by the direct arrivals and surface-reflected arrivals, where the grazing angle from sources are large. This phenomenon is similar to the seasonal effects of SSP on reliable acoustic path and bottom bounce in the deep ocean [30].

**Figure 12:**
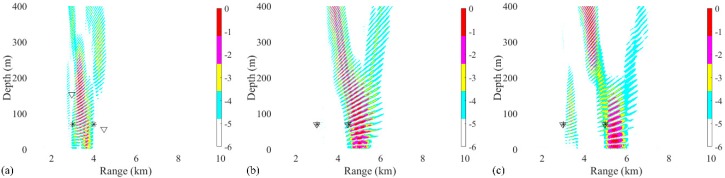
Ambiguity surfaces generated with 28 snapshots and a 32 element VLA using the RAMPMF method to localize sources at 70 m. The position of one source (3 km, 70 m) remains unchanged. The distances between two sources are (**a**) 1 km, (**b**) 1.5 km and (**c**) 2 km. True source positions and the positions estimated by RAMPMF methods are represented as black asterisks and inverted triangles, respectively.

In addition, the distance between two sources at a depth of 70 m also has influence on the performance of the RAMPMF method. The distances were chosen between 1 km, 1.5 km, and 2 km. The corresponding localization results were shown in Figure 12. It could be indicated from Figure 12a−c that the source positions could be estimated when the distance is greater than 1 km.

**Figure 13:**
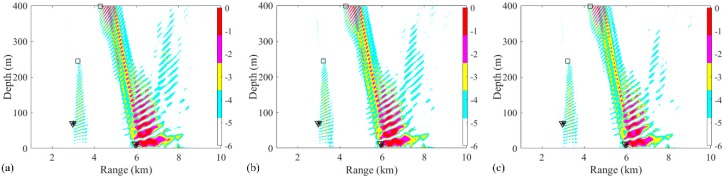
(**a**–**c**) Ambiguity surfaces generated by different realizations with 28 snapshots and a 32 element VLA using the RAMPMF method to localize sources (3 km, 70 m) and (6 km, 10 m). True source positions and the positions estimated by the RAMP and RAMPMF methods are represented as black asterisks, squares, and inverted triangles, respectively.

**Figure 14:**
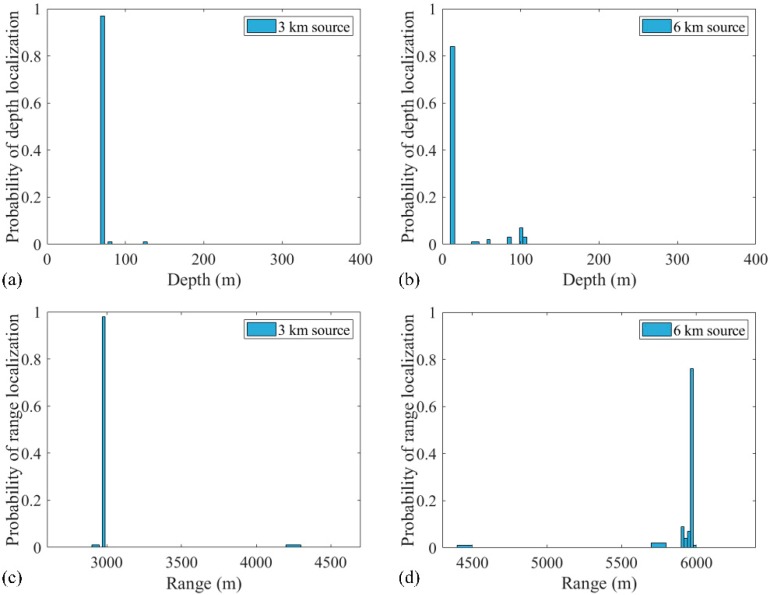
The probabilities of depth estimation and range estimation within 500 realizations for (**a**,**c**) the 3 km source and (**b**,**d**) the 6 km source.

The same processing was applied in the same environment, except that the source positions were (3 km, 70 m) and (6 km, 10 m). The localization performances of processors within three realizations are presented in Figure 13. Obviously, the RAMPMF performs best. The solutions resulting from pseudo peaks were eliminated by utilizing the spatial filter recurrently. Also, 500 realizations for this scenario were applied with the RAMPMF method. The probability of accurate localizations for sources (3 km, 70 m) and (6 km, 10 m) were found to be 96% and 79.6%. Figure 14 presents the distribution graphs of the estimated depths for two sources. Compared to the localization of sources (3 km, 70 m) and (6 km, 70 m), the estimation probability of the 6 km source decreases because the strong interferences caused by a shallower source can appear in the deep region, as shown in Figure 13, thus affecting the localization performance.

### 3.2. Experimental Data

**Figure 15:**
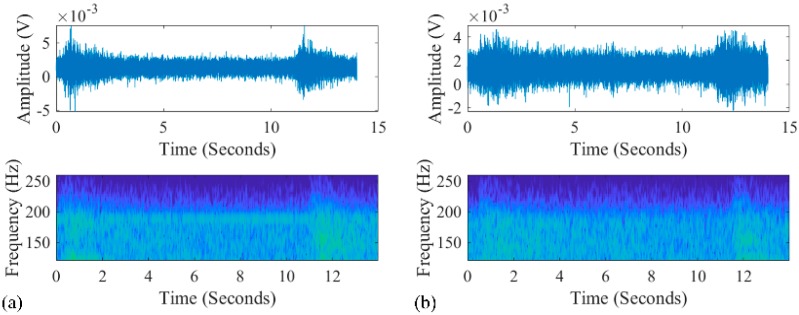
The signals received on the VLA at ranges of (**a**) 3.4 km and (**b**) 4.5 km. The time series between 0 and 2 s was received from an unknown broadband source.

The experimental data were sampled with a 32 element VLA with a sample rate of 4 kHz and a sensitivity of −180 dB re 1 V/µPa. Each hydrophone was treated as a Data acquisition channel of a quad, 24-bit, delta-sigma analog-to-digital converter. The data was saved to a 256 GB secure digital card. And this hydrophone was rated for operation from 5 Hz to 20 kHz. The hydrophones were deployed from 1735 to 1859 m with a 4 m interval. A source at frequency 187 Hz was towed by the research ship. The bathymetry of this region was approximated to be flat at a depth of 2000 m. The towed source was at depth of 100 m, and the corresponding Global Position System (GPS) positions were recorded. Two signals, which were transmitted from the towed source at different ranges (3.4 and 4.5 km) from the VLA, were chosen to demonstrate the performance of the RAMPMF method, as shown in Figure 15a,b. The unknown broadband interference source can be clearly observed. Therefore, the signals between 4 and 10 s (6 s in length) were used for processing. The received signals at one hydrophone are shown in Figure 15a,b. And the noise was chosen at the time intervals without the signals transmitted from the towed source. The corresponding SNRs were 6 and 0 dB, respectively. A Fast Fourier Transform (FFT) length of 4096 samples with 50% overlap generated 11 snapshots, resulting in time windows with a length of 2 s and a frequency resolution of approximately 0.5 Hz. The Kaiser window with β=2.5 was applied to each snapshot. These signals were combined artificially for demonstrating the performance of three methods.

**Figure 16:**
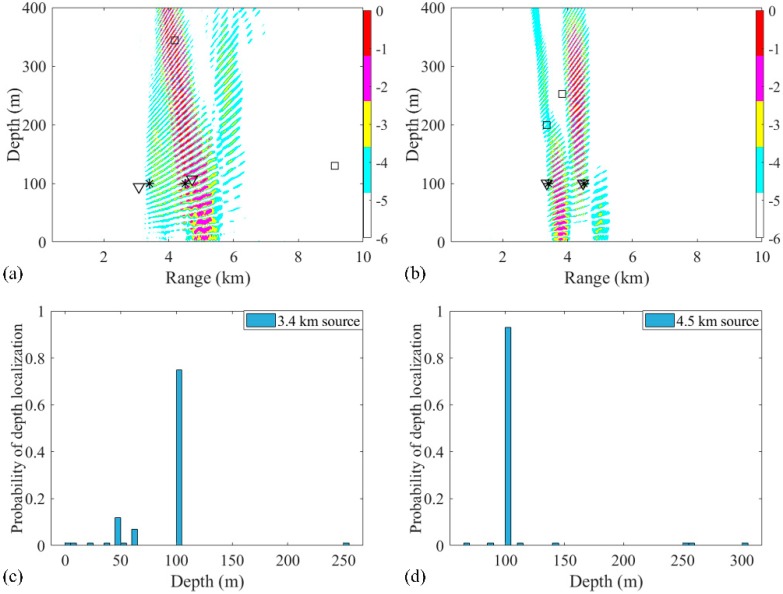
Ambiguity surfaces generated by (**a**) the experimental data and (**b**) the simulated data using the RAMPMF method to localize sources (3.4 km, 100 m) and (4.5 km, 100 m). True source positions and the positions estimated by the RAMP and RAMPMF methods are represented as black asterisks, squares, and inverted triangles, respectively. The depth distributions in 500 realizations of simulated data for (**c**) 3.4 km and (**d**) 4.5 km sources.

The source localization results by three different processors using the experimental data are shown in Figure 16a. Simulation of this scenario was also carried out. The positions estimated by RAMPMF were (3.08 km, 94 m) and (4.72 km, 107 m), respectively. As can be seen from Figure 16a,b, the RAMPMF method performs best in this scenario. The range and depth relative errors of the two sources were (9.4%, 6%) and (4.9%, 7%), respectively. The localization result and depth distribution in 500 realizations are shown in Figure 16b,c, respectively. The RAMPMF method was found to display fewer ambiguous peaks than the other two processors, similar to the localization results. It is shown that the probability obtaining an accurately estimated source depth at a range of 4.5 km is higher than that at range of 3.4 km in Figure 16c. Moreover, it is indicated that the localization error decreases for the further source in both the experimental and simulated results when the sources were at depth of 100 m. This behavior is not consistent with the results of the sources at an isothermal depth (< 80 m). Possible reasons for this may be the uncertainty of environmental parameters, and the strong interference of two close sources.

**Figure 17:**
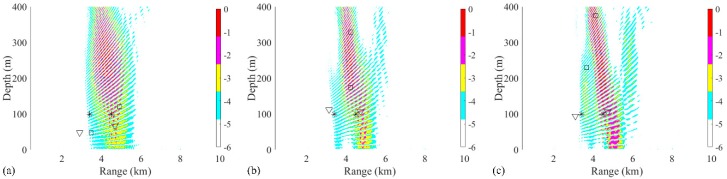
The ambiguity surfaces calculated by a Bartlett processor, the RAMP method, and the RAMPMF method with the apertures (**a**) 94 m; (**b**) 100 m; and (**c**) 112 m. True source positions and the positions estimated by the RAMP and RAMPMF methods are represented as black asterisks, squares, and inverted triangles, respectively.

**Figure 18:**
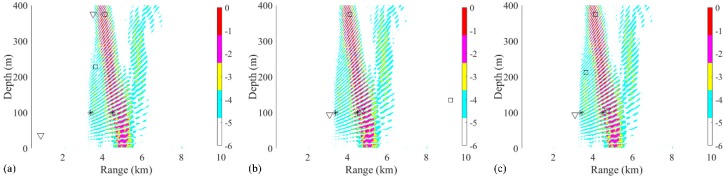
The ambiguity surfaces calculated by a Bartlett processor, the RAMP method, and the RAMPMF method with (**a**) 6; (**b**) 8 and (**c**) 10 snapshots. True source positions and the positions estimated by the RAMP and RAMPMF methods are represented as black asterisks, squares, and inverted triangles, respectively.

**Figure 19:**
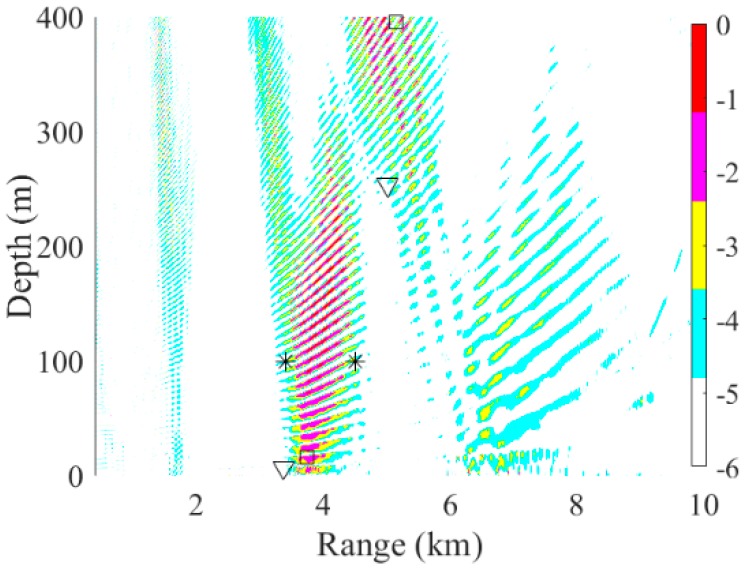
The ambiguity surfaces calculated by a Bartlett processor, the RAMP method, and the RAMPMF method with 8 m hydrophone spacing. True source positions and the positions estimated by the RAMP and RAMPMF methods are represented as black asterisks, squares, and inverted triangles, respectively.

Then, the effects of factors such as the array aperture, number of receivers, and number of snapshots on the localization with experimental data were also examined. First, the array aperture was chosen between 94, 100. and 112 m, and the localization results were shown in Figure 17. Similar to the results shown in Figure 17, the estimated source positions were close to the true source positions, when the aperture is larger than 100 m. Also, the number of snapshots was chosen between 6, 8 and 10 to estimate two sources positions, as shown in Figure 18. When the number of snapshots is more than 8, the localization results is as good as it using 11 snapshots. Finally, the hydrophone spacing was changed to 8 m, and the ambiguity surfaces is shown in Figure 19. It could be seen that the results were all far from the true sources. In summary, the effects influenced by the factors are consistent with them in the simulation.

## 4. Conclusions and Discussion

In this article, a new method (RAMPMF) for two-source localization for single frequency was presented. The method is based on a practical greedy algorithm in CS theory combined with a simplified spatial filter. Through searching the maximum value repeatedly, an iteration containing different source positions can be obtained. In addition, the corresponding estimation of sources are the positions in the iteration. The RAMPMF method was applied to the simulated and experimental data in a deep-water region with flat bathymetry, where the transmitting and receiving platforms were a single-frequency source and a 32 element VLA, respectively. And the performances of this method were demonstrated by the results of its application. Through the performances of this method, some conclusions can be summarized as follow:

(1) For the two-source scenario in the simulation, the proposed RAMPMF method was shown to localize two positions more accurately than the Bartlett and RAMP processors. Furthermore, a high resolution can be obtained in this method. The performance of the RAMPMF method was also demonstrated with the received data in the South China Sea, revealing that the estimated source positions are in good agreement with the depth of the towed source and the GPS measurements obtained in the experiment.

(2) The feasibility of the proposed method with a short array has been demonstrated by the impact of the array length on the localization performance. The probability of two-source localization above 0.9 can be achieved with the array length greater than 114 m, which is only 5.7% of the total water depth. On the other hand, the probability of depth estimation for an aperture less than 100 m is relatively low in the 2000-meter deep water. And the estimations of the ranges of sources are more accurate than the estimations of the depths. When the source depth is close to the sea surface at 6 km, a strong interference would be generated in the deep region. The interference could introduce pseudo peaks in the results and decrease the probability of accurate localization. Not surprisingly, the performance can also be improved by increasing the number of hydrophones used, widening the array aperture, utilizing a larger number of snapshots, and increasing the SNR. Besides, sources at a greater distance are localized with lower probability compared with sources close to the VLA. This phenomenon has been verified in both simulation and experiment.

(3) When the mismatched SSP is applied to calculate the replicas, the performance of the RAMPMF method will decrease. The degree of the influence by the mismatched SSP is not as much as it affected by SNR due to the physical characteristic of sound propagation from the surface source to the VLA at the bottom. In addition, the RAMPMF method cannot separate two sources at depth of 70 m, when the distance between them is less than 1 km.

(4) The high computation level, due to the large size of the sensing matrix with denser discretization in depth and range, can be decreased by the RAMP method compared to the SAMP method [27]. Also, a lower-rank projection matrix is used to construct the spatial filter more quickly compared to the conventional solution for a semidefinite program (SDP) [14]. Therefore, two-source localization can be completed more quickly by the RAMPMF method, compared to the SAMP method with a spatial filter obtained by CVX toolbox.

However, the proposed method may not be applicable to all situations. The main limitations and future studies about the proposed method are shown below:

(1) The major application of the proposed method is to localize a source of interest masked by a strong interfering source, when only a short array can be used in the deep ocean. The assumption is that two sources exist in the region of interest. It needs be improved when the number of sources changes. For one-source, the performance of the RAMP method based on compressive sensing in this paper is the same with the performance of the Bartlett processor. The proving process can be found in the appendix in reference [18]. For three sources or more sources, the proposed method needs be improved to localize all the targets. If the method is applied into a three or more sources scenario, the results would still be two estimated positions. Therefore, a constraint for the output power in each iterative may be used to improve the proposed method for a multiple sources scenario.

(2) Due to the ambient noise in the ocean, a very difficult problem to solve, the most common use of noise in the source localization problem is the white noise in underwater acoustics. The parameters affecting the performance of the proposed method, such as the aperture, SNR, and so on, has been demonstrated in this paper. It can be inferred that this method is a high-resolution algorithm with a high SNR and a large enough aperture. Therefore, effectiveness of the proposed method may decrease a lot when the noise is closely related to the signals.

(3) The proposed method is not a real-time processing algorithm. Additionally, it needs the replicas calculated by Bellhop which needs a lot of time. Therefore, a complexity study is not conducted but the complexity of the CS algorithm used in this paper is presented in the below references.

Further work is needed to fully understand the implications of the proposed method in the deep ocean. Analysis would be helpful for extending the proposed method to improve its robustness when taking into consideration the mismatch. Future studies should focus on the complexity of the improved method for real-time processing in practice.

## Figures and Tables

**Table 1:** The estimated positions using the RAMP method with a spatial filter in each loop.

Number of Loops	1	2	3	4	5	6
Range (m)	6920	2780	5960	2980	5920	2980
Depth (m)	49	32	70	70	69	70
